# Learning to Predict Ischemic Stroke Growth on Acute CT Perfusion Data by Interpolating Low-Dimensional Shape Representations

**DOI:** 10.3389/fneur.2018.00989

**Published:** 2018-11-26

**Authors:** Christian Lucas, André Kemmling, Nassim Bouteldja, Linda F. Aulmann, Amir Madany Mamlouk, Mattias P. Heinrich

**Affiliations:** ^1^Institute of Medical Informatics, University of Lübeck, Lübeck, Germany; ^2^Graduate School for Computing in Medicine and Life Sciences, University of Lübeck, Lübeck, Germany; ^3^Department of Clinical Radiology, University Hospital Münster, Münster, Germany; ^4^Institute of Neuroradiology, University Medical Center Schleswig-Holstein, Lübeck, Germany; ^5^Institute for Neuro- and Bioinformatics, University of Lübeck, Lübeck, Germany

**Keywords:** ischemic, stroke, prediction, growth, learning, shape, CT, perfusion

## Abstract

Cerebrovascular diseases, in particular ischemic stroke, are one of the leading global causes of death in developed countries. Perfusion CT and/or MRI are ideal imaging modalities for characterizing affected ischemic tissue in the hyper-acute phase. If infarct growth over time could be predicted accurately from functional acute imaging protocols together with advanced machine-learning based image analysis, the expected benefits of treatment options could be better weighted against potential risks. The quality of the outcome prediction by convolutional neural networks (CNNs) is so far limited, which indicates that even highly complex deep learning algorithms are not fully capable of directly learning physiological principles of tissue salvation through weak supervision due to a lack of data (e.g., follow-up segmentation). In this work, we address these current shortcomings by explicitly taking into account clinical expert knowledge in the form of segmentations of the core and its surrounding penumbra in acute CT perfusion images (CTP), that are trained to be represented in a low-dimensional non-linear shape space. Employing a multi-scale CNN (U-Net) together with a convolutional auto-encoder, we predict lesion tissue probabilities for new patients. The predictions are physiologically constrained to a shape embedding that encodes a continuous progression between the core and penumbra extents. The comparison to a simple interpolation in the original voxel space and an unconstrained CNN shows that the use of such a shape space can be advantageous to predict time-dependent growth of stroke lesions on acute perfusion data, yielding a Dice score overlap of 0.46 for predictions from expert segmentations of core and penumbra. Our interpolation method models monotone infarct growth robustly on a linear time scale to automatically predict clinically plausible tissue outcomes that may serve as a basis for more clinical measures such as the expected lesion volume increase and can support the decision making on treatment options and triage.

## 1. Introduction

Cerebrovascular diseases, in particular strokes, are one of the leading global causes of death in developed countries (1). Acute stroke, which is usually caused by the blockage of cerebral blood flow due to a blood clot, is often diagnosed through CT or MR perfusion imaging (beside others, such as CTA). The derived perfusion parameter maps, e.g., Cerebral Blood Volume (CBV) or Time To Drain (TTD), provide spatio-temporal distributions of a contrast medium bolus within brain tissue. In contrast to native CT or standard MR sequences, such as T2 or FLAIR, perfusion images with their apparent functional signals enable the delineation of the potential infarct area even in the early acute phase and allow to visually assess the expected stroke severity, which helps the radiologist to come to a final therapy decision as early as possible.

In order to decide for a treatment the doctor has to weigh the risk of a therapy such as thrombolysis or thrombectomy against the expected outcome. For instance, (2) describe hemorrhages, such as symptomatic intracerebral hemorrhage, as a typical risk of intravenous thrombolysis therapy. For large vessel occlusions, mechanical thrombectomy improves functional outcomes but is logistically challenging. It is of major importance to consider the immediate availability of a therapy option, since the expected outcome strongly relies on the onset-to-treatment time (3). Depending on the expected time until revascularization, the radiologist has to estimate if further progression of the stroke can be avoided so that substantial parts of the tissue-at-risk within the penumbra could be salvaged. In the infarct core, however, as evident by a decrease in CBV, severe tissue injury and permanent vascular collapse have occurred.

Since stroke lesions vary widely in shape or size, and also evolve spatially heterogeneously over time, it is challenging for the radiologist to estimate growth or the size of the potentially stroke-affected tissue. For this reason, it is difficult to derive a time window in which a specific therapy path may be beneficial over another. Deep learning with CNNs has become popular in medical image analysis over the past recent years by clearly exceeding the so far state-of-the-art results, potentially capable of modeling this complex relationship.

### 1.1. Objective

We present a novel tool for automatic stroke tissue outcome estimation using a CNN with a convolutional auto-encoder (CAE) that incorporates learned stroke shapes of core and penumbra. In a proof-of-concept, the trained model is able to predict the stroke lesion growth for patients with successful recanalization based on a given time-to-treatment for the thrombectomy (Table 1) and the CTP imaging parameter maps CBV and TTD. An evaluation of the method shows the practicality in principle on a limited dataset and the discussion provides pros and cons that suggest to further investigate this approach for clinical use.

**Table 1:** Inclusion criteria of the dataset for evaluation.

**Inclusion criteria**
Initial CT perfusion imaging
Thrombectomy
Follow-Up CT within 6–24 h
Age at least 18 years

### 1.2. Outline

In order to gain a fundamental understanding of the method and its design choices, we provide a methodological overview in the following section: First, a review on the established stroke image analysis methods in clinical research literature is given; Second, the foundations of different image representations that can help solving higher-level tasks for image analysis as well as machine learning methods that have been investigated for stroke imaging are described; Third, we explain the use of CAEs for regularization in image segmentation by learning shape representations and how our work is based upon it. Subsequent, a detailed description of the assumptions and components of our method is provided—this third section also explains how to reproduce the method, that is, how to train the shape space and predict follow-up lesions based on noisy shape estimates. The fourth section lists the materials for a comparative evaluation and discusses its results, before we provide a final conclusion in the last section.

## 2. Image analysis

Classic thresholding methods for stroke image analysis have the drawback of only modeling a single univariate hard decision border between affected and non-affected tissue. Even when splitting into subgroups of different admission times (4) or distinguishing core against penumbra, the result will be a binary map of the affected vs. unaffected tissue. Further, purely voxel-based methods can produce irregular and physiologically implausible shapes. Statistical models, e.g., a linear regression model as used by Kemmling et al. (5), can cope with the variances in the data according to the complexity of the model and proper parameterization, while simple models will usually show a strong bias when used for high-dimensional problems.

### 2.1. Representation and spaces

In general, noisy images make it difficult to operate in image space, for instance, to apply a threshold to images for extracting regions that define the outcome. The input representation is not always suitable to detect the complex input patterns that determine the output. As known from signal processing and analysis, transforming the input into another representation (extract *features*) can often make it easier to perform classification or regression tasks. There are many transformations, e.g., non-linear kernel methods can bring a representation into a higher dimensionality where it may become linear separable. As this is a vast field that shall not be described here in detail, we emphasize that the input image data often needs to be fit into a regularized model or transformed to another representation first, on which the high-level task becomes easier to solve.

Kolouri et al. (6) propose *transport spaces* based on optimal transport theory to model biomedical problems such as tumor growth. The idea of looking at images as mass particle distributions is related to tissue distributions in biology, for example, when learning the transformations of some sample images onto a mean non-pathological image to extract the main modes of variance ideally representing the change from benign to pathological tissue (7). The modes could be extracted by principle component analysis (PCA) or other machine-learning approaches, e.g., auto-encoders (8). The formulation of the transport space and described applications suggest to make use of it for modeling other biological growth processes. However, to our knowledge, this has not yet been investigated for the cerebrovascular domain and applicability remains unclear for stroke tissue prediction.

While for all the above-mentioned methods their transformations to acquire a new representation are predefined, there are other models whose parameters can be estimated from samples. For instance, this could be learning a statistical distribution, like the shape and appearance of point representations (9), where the parameterization of the probabilistic distribution is learned from a training set. With a suitable representation at hand, there are several ways to machine-learn rather than fitting the input representation with its outcome into a statistical model. This often leads to more accurate results: Before Deep Learning has become popular in the last years, the medical image community had investigated Decision and Regression Forest models extensively, and they have shown good performance over statistical linear models or boosting approaches (10). However, these methods rely on previously specified or separately learned feature representations that need to be extracted from the image data first.

Opposite to the prior definition of the representations used, one can also machine-learn the representation *and* the classification (or regression) both at once using non-linear artificial neural networks that are capable of learning sufficiently complex models without the need of tuning the right parameterization by hand. The review paper of Lee et al. (11) shows that the methods used with deep learning are still new to the field of stroke imaging and analysis. Some attempts with other machine learning methods have been made for diagnosis and prognosis, however, those models usually predict disease scores or specific clinical outcomes but not tissue outcome.

### 2.2. Deep learning for stroke imaging

Deep Learning with artificial neural networks is based on the idea of perceptrons where the output of a perceptron is computed by the weighted sum of its inputs *x* followed by an activation function σ (e.g., rectifier as in Equation 1). The power of such networks has been proven in the early work of Hornik et al. (12) by the fact that even a single hidden layer perceptron network with a proper activation function is capable of approximating any mathematical function. However, estimating that relation between input and output requires a lot of data and proper regularization since we have an underdetermined system when learning the coefficients *w*_*i*_ (neuron weights for incoming connections), otherwise.

(1) $$\begin{array}{r} z=\sigma \left(\underset{i}{\sum }w_{i}x_{i}\right),\;\sigma \left(y\right)=max\left(0,y\right) \end{array}$$

Although known for a long time, training and regularization of such networks is difficult. As a consequence of this, there have only been few attempts to utilize them for spatial data such as medical images, e.g., as proposed by Huang et al. (13) for predicting tissue fates of stroke on acute image data, but their performance could not be tweaked to exceed other former state-of-the-art approaches. For image data, the breakthrough came with deep (i.e., many layers) CNNs automatically learned through the back-propagation algorithm. Their layers form a feature hierarchy of increasingly complex features detected by the single layers through convolving the input of shared-weights kernel neurons, which themselves can be simulated by perceptrons. Interspersing pooling layers with spatial strides allows to learn texture or in general global features of the input images. See Schmidhuber (14) for an accurate explanation of these principles.

One of the first approaches modeling stroke tissue outcome with a deep learning CNN has been presented by Stier et al. (15). They trained a 2D-patch-based architecture with respect to the *T*_*max*_ feature from MR perfusion observed for acute ischemic stroke patients and a follow-up segmentation on FLAIR about 4 days later. The patch-based method clearly outperformed voxel-based approaches. As with other typical *black box*-like deep learning models, there are no further hyperparameters or constraints that can be set to control the prediction, e.g., for estimating the effect of time.

There are two major challenges in deep learning for stroke analysis tasks with regard to the data: First, there exists a general lack of accessible medical (ground truth) data and, second, the data is of irregular temporal nature. That makes it difficult to apply regular sequence models, such as Markov chains, recurrent neural networks, or the Long-Short-Term-Memory of Hochreiter and Schmidhuber (16). The data is temporally scattered: The points in time *t*_Onset_, *t*_Imaging_, and *t*_Treatment_ are sampled as patients rush into the hospital's emergency room and cannot be collected in a regular manner.

#### 2.2.1. U-Net architecture

The U-Net architecture of Ronneberger et al. (17) has been successfully and widely used for biomedical applications by producing semantic segmentations through a fully-convolutional CNN (18) that additionally incorporates skip connections between the context encoding and the refining decoding path for each scale level. The encoding-decoding pattern has established well especially for fully-convolutional networks and is also known from auto-encoders, as used in our proposed method of this paper. Considering different scales is usually a good approach to capture context and details, and this works already well with just two pathways as in the DeepMedic architecture of Kamnitsas et al. (19) who won the sub-acute ischemic stroke lesion segmentation task of the first ISLES challenge in 2015 (20).

At the 2017 edition of the ISLES challenge (21) we presented a robust network on perfusion image data to predict an average lesion outcome and ranked second overall for the binary segmentation output. Many of the top-ranked methods exploited a U-Net architecture, such as the challenge winner (22) who used a 3D U-Net within an ensemble along with other networks and focused on its hyperparameter optimization. In our 2D network instead, we added further skip connections within the encoding path to enhance sensitivity in particular for the difficult smaller lesions in comparison to a standard U-Net (23).

We did not observe advantages when providing clinical variables (e.g., disease scores, time points) as constant input features along with the perfusion images to predict the follow-up lesion, although they are known to be good predictors for the outcome. In fact, the 2D U-Net performance on the ISLES data could not benefit from the additional information and so we only used spatial perfusion maps to train on. The visual results suggested that rather the robust image features for detecting some highly probable necrotic stroke tissue were learned (cf. also our experiments later in this paper: Table 4, Figure 9). This makes such a network suitable for segmenting present perfusion lesions, but requires a new strategy to make use of clinical variables for predicting follow-up lesions. It remains an open question in literature, how to ideally incorporate clinical data in a U-Net-only architecture.

#### 2.2.2. Biomedical shape regularization

**Figure 1:**
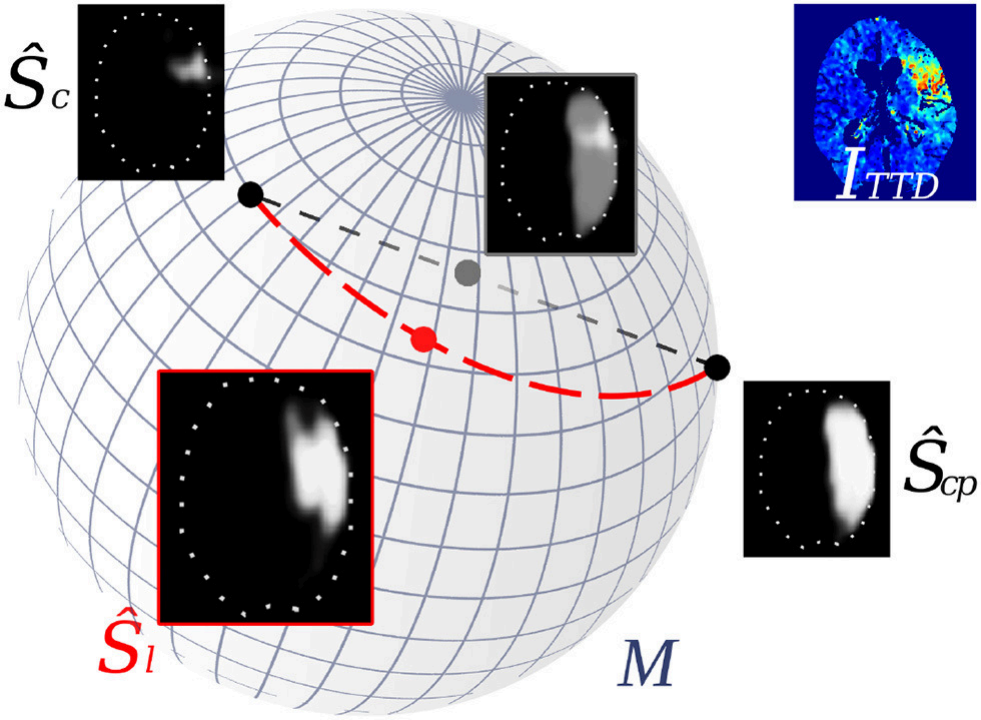
Interpolation of stroke shapes Ŝ_*c*_ and Ŝ_*cp*_: The trivial linear interpolation (gray line) in voxel space Ω leads to a fade-in/fade-out appearance of the two shapes. Embedding shapes non-linearly on a manifold *M* allows geodesic interpolation (red line) on *M* which results in a non-linear interpolation of the shapes in voxel space (Ŝ_*l*_).

Auto-encoders (AE) are one of several unsupervised methods to learn meaningful features from a data representation by typically encoding the input data *x*∈Ω into a lower-dimensional representation (*bottleneck*) and decoding this representation to get the reconstruction *z*∈Ω of the input *x* (Equation 2). This can be achieved through classical fully-connected layers or also by shared-weights convolutional layers for image data. In a convolutional auto-encoder (CAE) as introduced by Masci et al. (8) encoder *E*(*x*) usually consists of a typical convolutional feature hierarchy (akin to CNNs) that results in a discriminative latent code *y*∈*M*, which could be a feature vector or map. Decoder *D*(*y*) computes a reconstruction *z* back in input space Ω. During training, the weights of both are optimized such that a loss *L*(*x, z*), e.g., the mean squared error $\frac{1}{n}\underset{n}{\sum }{\left(x-z\right)}^{2}$ for *n* training samples, is minimized. If used with volumetric segmentations, one can learn shape embeddings on a low-dimensional manifold *M* (Figure 1) with its dimensions representing some main modes of the shapes by optimizing the CAE:

(2) $$\begin{array}{r} z=\left(D{}^{\circ}E\right)\left(x\right). \end{array}$$

The principle of shape-constrained segmentation learning was proposed by Ravishankar et al. (24), whose cascaded architecture includes a U-Net and a CAE for shape regularization. While the U-Net follows the same encoder-decoder principle like the convolutional auto-encoder, it does not learn local geometry and shape but produces rather noisy predictions through its skip connections that *skip* its inner bottleneck. The authors combine both sub-tasks of segmentation and reconstruction in an overall loss to utilize the anatomically regularizing bottleneck of the auto-encoder for completing noisy kidney segmentations, which improves the segmentations by about 5% compared to U-Net only.

Oktay et al. (25) presented an anatomically constrained neural network (ACNN) approach to also incorporate shape constraints of anatomical labels as prior knowledge. Their generic training scheme can be applied to various image analysis tasks and was documented for image segmentation and super-resolution. By using a CAE that is trained on ground truth shapes, they constrain the predicted image segmentations to lie close to the learned latent representation of the ground truth. In the end the decoder produces an anatomically constrained reconstruction of the segmentation from the learned shape space, because the segmentation has indeed been forced during training to lie close to the anatomy shape ground truth.

### 2.3. Our contribution

In this paper – based on the robust results that U-Nets achieve on perfusion imaging data and the shape-constrained network idea of Ravishankar et al. (24)—we present a novel methodology that:

1. Utilizes a 3D U-Net and constrains its segmentations on the CBV and TTD maps through a 3D CAE;
2. Enables continuous interpolation within the non-linear and low-dimensional embedding of both core and core+penumbra segmentations according to the time-to-treatment, that results in a shape-constrained prediction of the final lesion;
3. Empirically demonstrates—quantitatively and visually—the feasibility of predicting the final lesion shape from core and core+penumbra segmentations, as well as the advantage over using an unconstrained CNN or linear interpolations in image space.

## 3. Methods

Our idea is to estimate a time-to-treatment-dependent tissue outcome based on CBV and TTD perfusion images. We hypothesize that the minimum and maximum extents of the potential final stroke lesion can be approximated by delineating the core and penumbra area on the perfusion maps. This includes the following assumptions:

1. The CBV is sufficient for segmenting the core area, and TTD for core+penumbra.
2. The final lesion extents will not exceed the area boundaries of the tissue-at-risk outlined by the penumbra, but continuously evolve from the necrotic parts of the core in the direction of the outer penumbral boundary.
3. Growth over time is conducted linearly in the non-linear shape space.
4. We set a limit of 10 h after stroke onset for the infarct progression to reach the final size of core+penumbra. This value is chosen by experience and technically has to contain the maximum time-to-treatment from all training samples (maximum in our evaluation dataset: 7 h after onset).

It should be noted that training a model normalized to the acute stroke phase time range of 24 h is possible (cf. results of our evaluation later in this paper: Table 3, Figure 8) and recommended, if enough follow-up lesion data is available to sample roughly the entire space between core (0 h) and core+penumbra (24 h) representations to avoid areas of uncertainty.

### 3.1. Architecture

**Figure 2:**
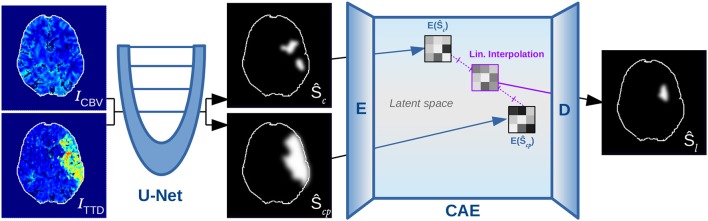
Overview of proposed method, showing the U-Net for segmenting core (Ŝ_*c*_) and penumbra (Ŝ_*cp*_), which are forwarded to the encoder *E* of the CAE for transforming them into low-dimensional shape space representations such that they can be linearly interpolated and decoded to output a follow-up estimate Ŝ_*l*_.

The method consists of a two-phase neural network that combines three main components for automatic shape-constrained follow-up lesion prediction (Figure 2):

1. U-Net estimating core (Ŝ_*c*_) and core+penumbra (Ŝ_*cp*_) from CBV and TTD maps.
2. CAE transforming the segmentations into a shape space and back.
3. Linear interpolation in the shape space to predict the follow-up lesion (Ŝ_*l*_).

First, the perfusion images *I*_*CBV*_ and *I*_*TTD*_ are processed by a U-Net *U* to compute the segmentation estimates Ŝ_*c*_ and Ŝ_*cp*_. Second, the encoder *E* of the CAE transforms each segmentation into a low-dimensional shape embedding ŷ_*c*_ and ŷ_*cp*_ of a shape space that must be learned beforehand. Linear interpolation (ŷ_*i*_, Equation 3) between the latent core and core+penumbra codes ŷ_*c*_ and ŷ_*cp*_ is conducted according to the expected *t*_Imaging → Treatment_ time, which must be normalized by the remaining time to reach 10 h after onset (corresponding to the total core+penumbra).

(3) $$\begin{array}{r} {ŷ}_{i}={ŷ}_{c}+\eta \left({ŷ}_{cp}-{ŷ}_{c}\right),\;\eta =\frac{t_{\text{Imaging}\to \text{Treatment}}}{10-t_{\text{Onset}\to \text{Imaging}}} \end{array}$$

**Figure 3:**
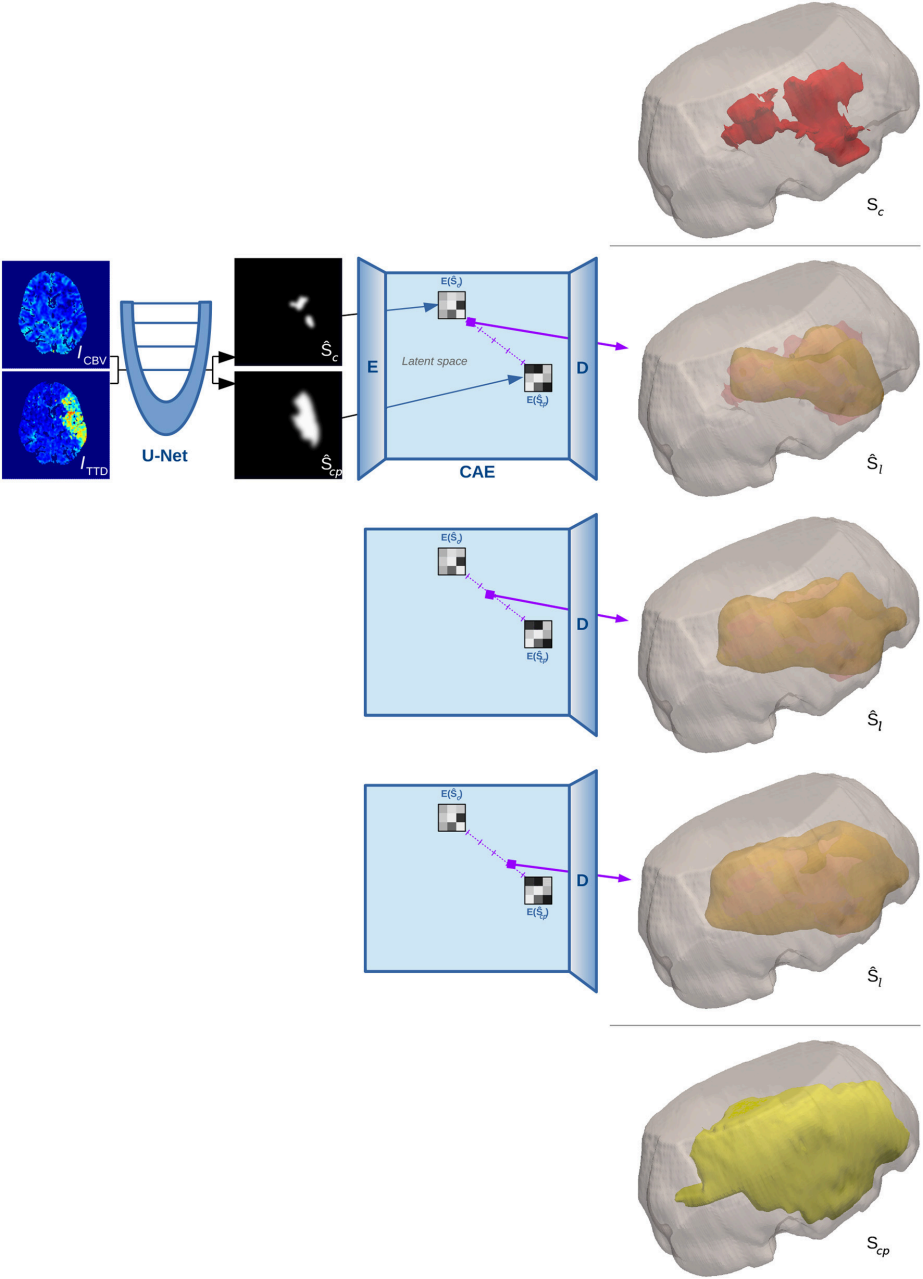
3D surface illustration for the binarized reconstruction of the interpolation in shape space for a single test case from our experiments. The top image shows the manual segmentation of core in red within a cutout of the brain volume, while the bottom image shows the ground truth segmentation of penumbra in yellow. Three steps of the interpolation between the estimates of both manual segmentations are shown in the middle rows.

This linear interpolation in shape space is crucial, as it corresponds to a non-linear interpolation of the reconstructed shapes on the manifold (Figure 1). The decoder *D* of the CAE is required to compute that reconstruction of the interpolated code ŷ_*i*_ in the voxel space with a segmentation Ŝ_*l*_ for the final lesion as result (Figure 3 illustrates a binarized 3D segmentation).

#### 3.1.1. Cascading networks for prediction

The construction of a two-phase network targets different sub-tasks that constrain the learning of the high-dimensional and complex overall task of follow-up lesion prediction. When discriminating only the final lesion binary label from background through high-dimensional multivariate input, it makes it hard for a machine learning algorithm to properly generalize the relation between input and outcome. Instead of directly estimating a follow-up segmentation from the input data, we guide the first sub-network (U-Net) to segment core and penumbra correctly, which is—as explained before—of major importance for predicting final lesion tissue outcome. Once this data is provided, the second sub-network (CAE) can learn the most salient shape features on a rather simplified representation with respect to the task (shape probability maps vs. different physiological CTP parameters) along with clinical data to estimate the follow-up lesion.

#### 3.1.2. U-Net

Instead of taking the 2D U-Net that we have used before at the ISLES 2017 challenge, we employ a smaller standard 3D U-Net *U* to reduce computational and memory demand while it can cope well with the three-dimensional nature of stroke volume data. Furthermore, instead of forwarding the full 128 × 128 × 28 input CTP images the U-Net is trained on randomly positioned cubic patches of size 64 × 64 × 28 (with additional padding of 20 voxels in each direction) and thus needs to forward 4 patches for segmentation of one single case. It receives patches from *I*_*CBV*_ and *I*_*TTD*_ as input and estimates Ŝ_*c*_, Ŝ_*cp*_ = *U*(*I*_*CBV*_, *I*_*TTD*_). The U-Net is build of double-convolutional blocks as known from Ronneberger et al. (17), while each 3 × 3 × 3 convolution is preceded by a batch normalization layer of Ioffe and Szegedy (26) for data whitening. The blocks are spread over three resolution levels (two Max-Poolings) with 16, 32, and 64 channels, respectively. This sums up to a total of about 355.000 network parameters.

#### 3.1.3. Convolutional auto-encoder

**Figure 4:**
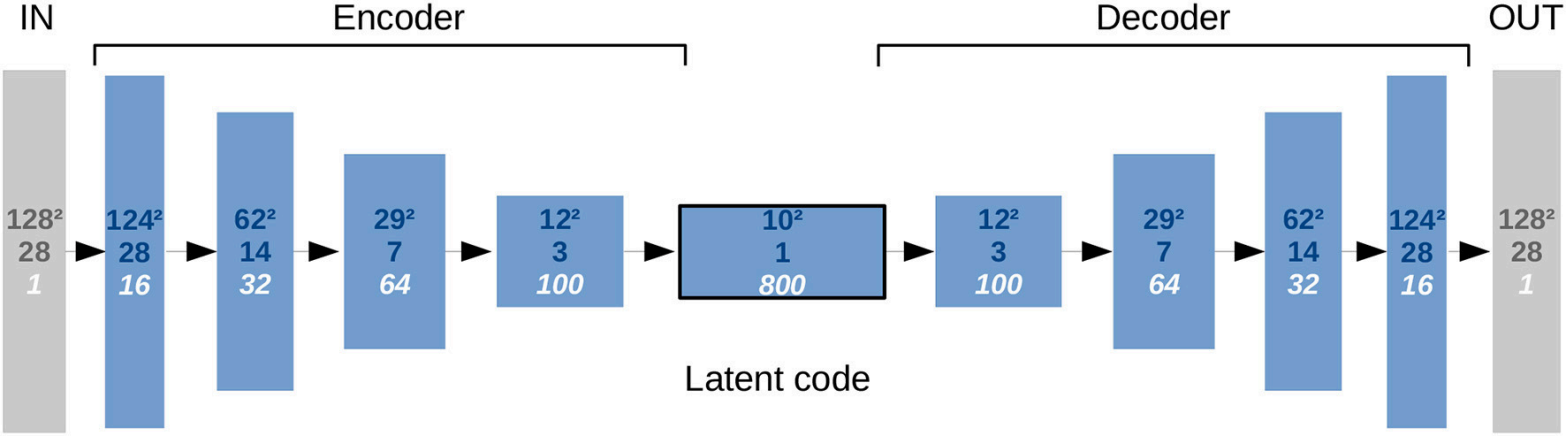
Representational complexity of the CAE and reduced dimensionality in the bottleneck. The numbers given top to bottom indicate: Quadratic size of the first two spatial dimensions, size in the third spatial dimension (axial), and number of feature channels. There is one input channel for the segmentation image and one output channel for its reconstruction.

Focusing on the subsequent CAE, we had to ensure some minimum number of layers (Figure 4) to detect the most salient and descriptive abstract stroke shape features while being well-regularized in order to reconstruct a good general estimate of the shape. This requires a bottleneck layer between *E* and *D* to produce low-dimensional latent codes that must have a limited but sufficient dimensionality. Consequently, only the main modes should be represented in the code with their major variances describing different stroke shapes without noise or overfitting of training samples. Regarding overfitting, our linear approach for the interpolation on the low-dimensional codes is in principle also robust against noise in time.

The input of the CAE is forwarded akin to the U-Net through double-convolutional blocks including batch normalization layers. Instead of two Max-Pooling operations, three additional 2-stride convolutional layers intersperse those blocks, while the final block is a single 3 × 3 × 3 convolution that convolves the feature map to the 10 × 10 × 1 bottleneck size. Like for the U-Net, 3 × 3 × 3 filter kernels throughout all convolutional layers of the CAE, as introduced by Simonyan and Zisserman (27), are exclusively used to decrease complexity compared to networks with bigger kernels while the same receptive field sizes can be reached by just stacking more layers of the smaller kernels. This results in more non-linearities and usually generalizes better.

The decoder is a mirrored encoder, with deconvolutional layers replacing the 2-stride convolutions. With this architecture, a single 128 × 128 × 28 shape segmentation image can be encoded into a low-dimensional code representation, and then decoded back to a segmentation image. Note that although the U-Net is fed with both CBV and TTD images as separate channels at once, the CAE's encoder has to forward their segmentations independently to get two separate latent codes, which will be then interpolated and forwarded in one step through the decoder to get the final lesion prediction.

Since there are several global scalar predictors (time, age, sex, clinical scores) that might be required to model the space properly, we also tried to map the shape segmentation input of the CAE to a vector representation (instead of spatial feature maps) in the bottleneck by using a fully-connected or convolutional layer with appropriate kernel size. This would have allowed us to add an arbitrary number of scalar values directly as additional dimensions to the latent code in the bottleneck and to easily quantify the dimensionality of such shape representations in the latent space. However, regularization is difficult – even when using dropout (28)—and the reconstructions are less accurate than with latent spatial feature map representations. Therefore, the CAE remains convolutional-only and no clinical variables other than the combination η of both time predictors for the interpolation are used as per definition in Equation (3).

### 3.2. Training

**Figure 5:**
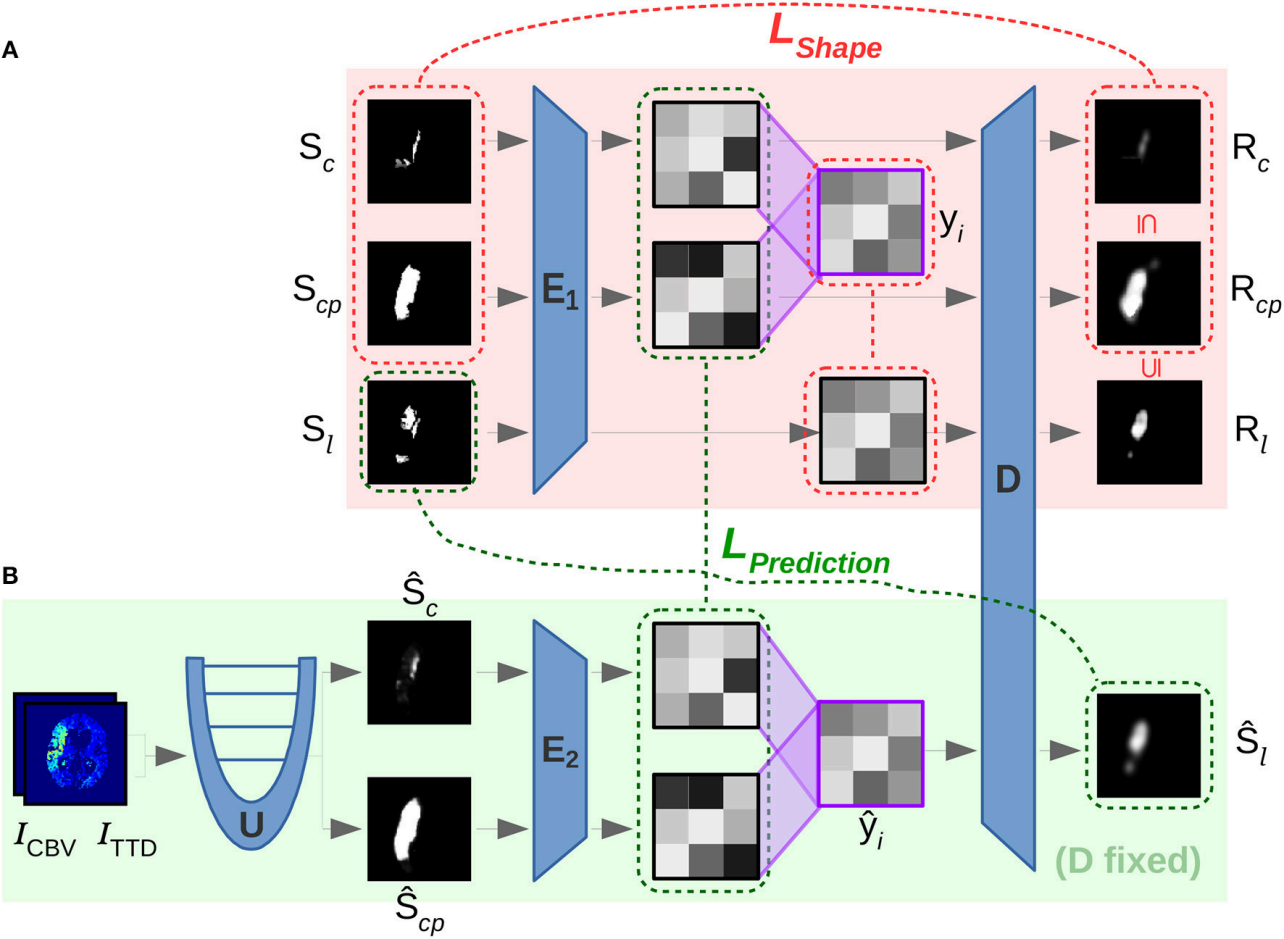
Overview of involved convolutional sub-networks (blue) in the two-phase training according to section 3.2 in the text. In the first phase **(A)** the shape space is trained with *E*_1_ and *D* by using loss *L*_*Shape*_ (red) to reconstruct *R*_*c*_ and *R*_*cp*_. In the second phase **(B)** *E*_2_ is trained to map segmentations from *U* into the shape space via the second loss *L*_*Prediction*_ (green). The latent code ŷ_*i*_ interpolated between ŷ_*c*_ and ŷ_*cp*_ is decoded with the previously trained *D* of phase **(A)** for predicting the desired follow-up lesion Ŝ_*l*_.

The training is conducted in three consecutive steps (see Figure 5 for illustration of steps 2 and 3) that are characterized by different objectives formulated in their corresponding losses:

1. Training the U-Net with a *L*_*SoftDice*_ loss.
2. Training *E*_1_ and *D* to learn the shape space of the CAE from manual segmentations (*L*_*Shape*_)
3. Training *E*_2_ to fit automatic segmentation estimates of *U* into the shape space (*L*_*Prediction*_)

The U-Net is initially trained beforehand using the stochastic gradient variant ADAM of Kingma and Ba (29) for optimization. The ground truth segmentations *S*_*c*_ and *S*_*cp*_ of core and core+penumbra are used to penalize their predictions with a bigger loss for less overlap in the SoftDice measure, which is defined for all voxel positions *i* in a segmentation *A* with ground truth *B* and a small constant ϵ as follows:

(4) $$\begin{array}{r} SoftDice\left(A,B\right)=\frac{2\cdot \underset{i}{\sum }\left(A_{i}B_{i}\right)+\epsilon }{\underset{i}{\sum }\left(A_{i}A_{i}+B_{i}B_{i}\right)+\epsilon } \end{array}$$

#### 3.2.1. Shape space learning

First, a low-dimensional shape space is learned that embeds the ground truth shapes of core and core+penumbra segmentations, *S*_*c*_ and *S*_*cp*_. This is enforced by loss *L*_*Shape*_ in Equation (5), that consists of three parts: (1) Reconstruction *R*_*c*_, *R*_*cp*_, and *R*_*l*_ of core, core+penumbra and final lesion shape, (2) the property of the reconstructed core/lesion volume to still be a subset of the core+penumbra volume, and (3) a *L*1 loss for keeping the latent code *y*_*l*_ of the lesion shape close to the linear interpolation *y*_*i*_ of the core and core+penumbra codes:

(5) $$\begin{array}{r} L_{Shape}=\underset{s\in \left\{c,cp\right\}}{\sum }L_{SoftDice}\left(R_{s},S_{s}\right)+\underset{s\in \left\{c,l\right\}}{\sum }L_{mono}\left(R_{s},R_{cp}\right)+\alpha L1\left(y_{l},y_{i}\right) \end{array}$$

For the first 25 epochs α = 0 holds, otherwise α = 1. We observed that this helps the non-convex optimization function to first learn to reconstruct the shape into the correct brain hemisphere and was found to robustly prevent the network from getting trapped in implausible minima at the beginning of the training. Since *SoftDice*∈[0, 1] with 0 indicating non-overlap and 1 for full overlap, we need to define *L*_*SoftDice*_(*S*, Ŝ) = 1−*SoftDice*(*S*, Ŝ). To force the CAE to learn that the interpolation is only growing when interpolating along the (time) trajectory from core until reaching the core+penumbra segmentation at maximum, we define a constraint *L*_*mono*_ for all voxel positions *i* in two segmentation images *A, B* so that the reconstructions of the core segmentation and the intermediate lesion interpolation are monotone increasing to the total core+penumbra segmentation:

(6) $$\begin{array}{r} L_{mono}\left(A,B\right)=\underset{i}{\sum }max\left(A_{i}-B_{i},0\right) \end{array}$$

#### 3.2.2. Noisy shape interpolation

In the second training phase, the encoder and decoder weights from the shape space learning phase before will be fixed. A second encoder is then learned for the U-Net predictions Ŝ_*c*_ and Ŝ_*cp*_ of core and core+penumbra to compute latent representations ŷ_*c*_ and ŷ_*cp*_ that are located close to the shape embeddings *y*_*c*_ and *y*_*cp*_ of the corresponding ground truth segmentations in terms of *L*1 norm. *L*_*Prediction*_ in Equation (7) further enforces the monotone properties for the reconstructed segmentations and the main goal of high overlap for the prediction Ŝ_*l*_ decoded from the interpolated code ŷ_*i*_ to the actual follow-up ground truth *S*_*l*_:

(7) $$\begin{array}{r} L_{Prediction}=L_{SoftDice}\left({Ŝ}_{l},S_{l}\right)+\underset{s\in \left\{c,l\right\}}{\sum }L_{mono}\left({Ŝ}_{s},{Ŝ}_{cp}\right)+\underset{s\in \left\{c,l,i\right\}}{\sum }L1\left({ŷ}_{s},y_{s}\right) \end{array}$$

This way, the decoder *D* of the first phase ideally decodes an approximate representation from the shape space so that the reconstruction of the core and core+penumbra estimates should be close to the ground truth reconstruction of core and core+penumbra, (*D*°*E*_2_)(Ŝ_*c*_)≈(*D*°*E*_1_)(*S*_*c*_) and (*D*°*E*_2_)(Ŝ_*cp*_)≈(*D*°*E*_1_)(*S*_*cp*_). Moreover, the main goal remains to achieve an interpolation as close as possible to the true lesion segmentation:

(8) $$\begin{array}{r} D\left(\eta \cdot E_{2}\left({Ŝ}_{cp}\right)+\left(1-\eta \right)\cdot E_{2}\left({Ŝ}_{c}\right)\right)\approx \left(D{}^{\circ}E_{1}\right)\left(S_{l}\right) \end{array}$$

The individual loss terms have not been weighted by further manual parameters as we found no benefit for other than the uniformly weighted individual loss parts for both *L*_*Shape*_ and *L*_*Prediction*_. Further, we tried to apply both losses beforehand in an alternating and joint manner but this did not let the optimizer find proper minima; in particular, learning the exclusive occurrence of a stroke on either hemisphere could not be learned, which is what the network basically learns during the first epochs of training.

## 4. Experiments

We run a 5-fold test on a 29 subjects dataset, because the time demand for a full cross-validation was too high. In order to test each of the 5 folds, we thus had to train 5 models with the remaining 4 folds. Four of the folds consist of 6, and 1 fold consists of 5 cases. About one fourth of the training samples were used as validation set, so that a training set consists of 17 or 16 cases and is validated in each epoch on 6 other cases (disjoint with the test fold).

We prevent overfitting of the model by training until the validation loss converges and choose the model with the lowest validation loss. Since the model is eventually tested on a different fold of patients not used for the training and validation, we also avoid that the evaluation results could be tuned on the validation loss optimum. Due to the huge number of parameters in our 3D sub-networks, the memory demand for gradient computation increases rapidly, so a batch size of 4 had to be chosen to fit the training data into 11 GB of GPU memory.

### 4.1. Data

We used a dataset of 29 subjects from the Neuroradiology department at the University Hospital Schleswig-Holstein formerly collected for the TRAVESTROKE project for which one rater had created manual segmentations on the CT perfusion (CTP) modalities CBV (Cerebral Blood Volume) and TTD (Time-To-Drain) at the time of admission for core and core+penumbra, as well as a lesion segmentation on follow-up CT after treatment. The data was acquired with a Siemens Somatom Definition AS 40 (Siemens Healthcare GmbH Forchheim, Germany) and the raw data was deconvolved using the vendor algorithm to get CT perfusion parameters such as CBV or TTD. All patients of the dataset had been treated successfully with thrombectomy (TICI score 2b or 3). See baseline characteristics of the subjects included in the evaluation in Table 2.

**Table 2:** Characteristics for subjects with manually segmented core, penumbra and follow-up lesion of the retrospectively collected data.

**Baseline characteristics**	**Value**
Subjects *n*	29
Male sex, *n* (%)	16(55%)
Age, years, median (IQR)	70(63−77)
Admission NIHSS, median (IQR)	15(12−16)
Core volume, ml, median (IQR)	27(3−86)
Penumbra vol., ml, median (IQR)	164(149−199)
Lesion (FU) vol., ml, median (IQR)	31(18−93)
*t*_Onset → Imaging_, hours, median (IQR)	1.7(1.4−3.4)
*t*_Imaging → Treatment_, hours, median (IQR)	1.7(1.4−2.1)
*t*_Onset → Treatment_, hours, median (IQR)	3.9(3.2−4.9)

The dataset was pre-processed with FSL-FLIRT (30) for affine registration to correct tilted heads and transform them into common space. A downsampling was applied so that input size of the CBV and TTD maps was 128 × 128 × 28 voxels for reducing the computational demand. Additional clinical data for each subject was given. For the evaluation we only used the two durations *t*_Onset → Imaging_ and *t*_Imaging → Treatment_, which were normalized according to Equation (3). All shape segmentations have been elastically deformed in each epoch to augment the limited available training data for learning generic features of the CAE such that the representations in shape space can be robustly reconstructed.

### 4.2. Comparison

In section 2.2.1, we referred to the ISLES 2017 task of predicting a follow-up lesion based on MR perfusion data. We participated in this challenge with a single U-Net directly predicting the final follow-up lesion, as many of the teams were using unconstrained CNNs [as presented in 21]. Since this does not lead to accurate predictions of a progressed stroke when facing acute image data, we compare our proposed method with this simple U-Net approach with and without clinical time points as input. Unfortunately, the ISLES dataset consists of MR perfusion data without appropriate core and penumbra segmentations, so we cannot directly compare on the same dataset.

Our sub-task of linearly interpolating along the trajectory between core and core+penumbra requires representations of such in a suitable non-linear shape space that has to be learned before. In order to show the advantage of conducting this in such a shape space, we compare with the naïve way of linearly interpolating the shape segmentations in Ω. This can be simply computed with the same η as defined before in Equation (3):

(9) $$\begin{array}{r} {Ŝ}_{l}={Ŝ}_{c}+\eta \left({Ŝ}_{cp}-{Ŝ}_{c}\right) \end{array}$$

## 5. Results

The reconstruction results (Table 3) demonstrate the capability of our learned model to make time-dependent predictions and present the advantages of our CAE approach using core and penumbra segmentations to generalize well even from a small training dataset to estimate non-linear follow-up lesion interpolations. A Dice overlap of 0.46 was achieved in comparison to a manual rater. Considering a reconstruction Dice overlap for the CAE itself of 0.68 and 0.90 for core and core+penumbra, respectively, this represents a good result. In a use case, where a clinical expert manually segments core and penumbra, this can already be a helpful estimate for assessing the expected treatment outcome after thrombectomy.

**Table 3:** Reconstruction (1. training phase) results: Average Dice values on the test data for the CAE trained on core (*S*_*c*_) and core+penumbra (*S*_*cp*_) expert segmentations, as well as average Dice overlap of the follow-up expert lesion segmentation (*S*_*l*_) with the reconstructed interpolation from shape space (*R*_*i*_).

**Method**	**Method**	**# Parameters**	**Dice**		
**Core/Penumbra**	**Lesion (Training phase)**	**of network**	**Core (*R*_*c*_)**	**Core+Penumbra (*R*_*cp*_)**	**Lesion (*R*_*i*_)**
*Expert*	*Oracle* CAE 10 h (1. phase)	4.7·10^6^	0.68	0.90	0.53
*Expert*	CAE 10 h (1.)	4.7·10^6^	0.68	0.90	0.46
*Expert*	CAE 24 h (1.)	4.7·10^6^	0.70	0.90	0.44

*While the first row shows an oracle prediction overlap for the theoretically best-fit per case interpolation within our model (could be before or after the true time-to-treatment), the second row lists the result for our proposed approach using the actual time-to-treatment ground truth to predict the follow-up lesion from ground truth. The third row indicates that the effect of choosing a different normalization value from within the time range of the acute stroke phase can rather be neglected*.

**Figure 6:**
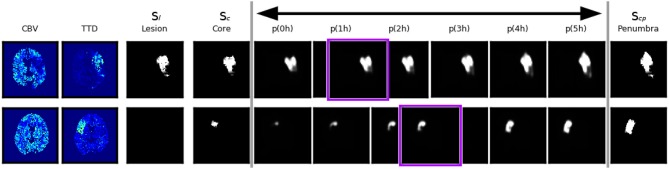
Two example cases with good and bad performance from the validation set at the end of the shape space learning (1. training phase) and their interpolation at different *t*_Imaging → Treatment_∈[0, 5] values. The first row shows an axial slice of an accurate reconstruction of core at 0*h* as well as of the penumbra, and as a consequence the non-linear interpolation of both gives a reliable estimation of the true follow-up lesion at its actual *t*_Imaging → Treatment_ value (outlined in purple). The second row shows a case where the temporal progression of the stroke is different, such that the final lesion was much smaller than a interpolation between core and penumbra would suggest.

However, even a very good reconstruction of core and penumbra does not guarantee a good final lesion estimate (Figure 6, bottom row). The quality and severity of the stroke in routine clinical data is not always fully encoded in its core and penumbra shape segmentations, and some of the follow-up lesion segmentations are actually smaller than even their corresponding core segmentations contrary to the definition that core should include only necrotic tissue which cannot be recovered. Potential reasons for this include the challenges of the manual annotations based on CBV and TTD alone.

With our trained model we could determine an upper bound of 0.53 for a linear interpolation-based lesion prediction *oracle* (Table 3), which does not use the true time-to-treatment but knows the correct η∈[0, 1] that results in the best overlap with the ground truth lesion. Apart from non-linear growth over time that has been observed in literature (31) and the lack of any information from the perfusion signal, the difference between 0.46 and 0.53 could be explained by too much noise in the time data; Especially the determination of *t*_Onset → Imaging_ can often be quite inaccurate in clinical practise. While it would be desirable to have the best interpolation reconstructed from times as near as possible to the true time-to-treatment, the linear approach is quite robust against inaccurate times and monotone growth is enforced. The doctor is essentially interested to see if there will be much relative growth and, consequently, how much of the tissue could be salvaged within the next hours.

Given the CAE, the second encoder *E*_2_ learns to map the segmentations from the U-Net with a high quality into the shape space during the second training phase (Table 4). This phase requires only 50 epochs for a convergence of the validation set loss, compared to 200 epochs in the first phase when training *E*_1_ and *D*. Automatic core+penumbra segmentations achieved by our U-Net model are of very high quality (Dice score of 0.81) and close to the optimal reconstruction given the constraints of the CAE (Dice of 0.90). The segmentation of the core is more challenging yielding Dice scores of only 0.45 which is improved by the second CAE to 0.55. This confirms the results of the shape-constrained network proposed by Ravishankar et al. (24).

**Table 4:** Experimental results from 5-fold test data based on the U-Net's Ŝ_*c*_, Ŝ_*cp*_, and lesion estimates: The average values for the Dice overlap with the ground truth segmentations *S*_*c*_, *S*_*cp*_, and *S*_*l*_ are presented.

**Method**	**Method**	**# Parameters**	**Dice**		
**Core/Penumbra**	**Lesion (Training phase)**	**of network(s)**	**Core**	**Core+Penumbra**	**Lesion**
U-Net	CAE (2. phase)	3.6·10^5^+4.7·10^6^	**0.55**	**0.81**	**0.43** (Ŝ_*l*_)
U-Net	CAE (1. phase)	3.6·10^5^+4.7·10^6^	0.43	0.80	0.40 (Ŝ_*l*_)
U-Net	Image Interpolation	3.6·10^5^	0.45 (Ŝ_*c*_)	**0.81** (Ŝ_*cp*_)	0.36
–	U-Net *2in*	3.6·10^5^	–	–	0.34
–	U-Net *4in*	3.6·10^5^	–	–	0.22

*The baseline U-Net-only approach has the same architecture as used in combination with the CAE above, but instead of predicting core and penumbra (2 channel output), it directly predicts the follow-up lesion (1 channel output) as described in section 4.2. We input CBV and TTD maps only (2in), or additionally both t_Onset → Imaging_ and t_Imaging → Treatment_ as constant image channels (4in), whereas the latter performed worse coincident with our previous experience (see section 2.2.1 and Figure 9). The highest overlaps for core, core+penumbra, and lesion are highlighted in bold*.

**Figure 7:**
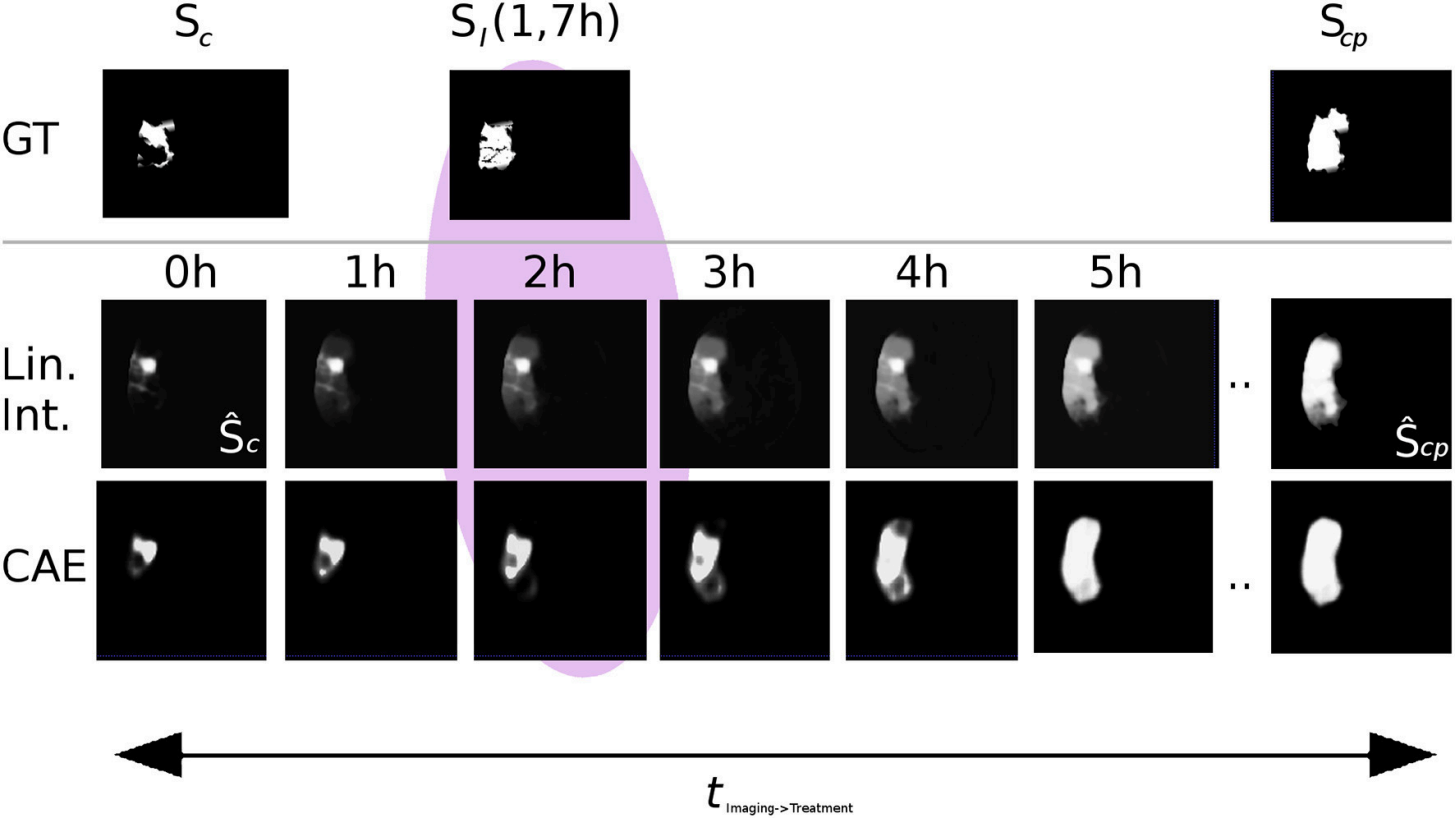
Visual comparison of linear interpolation in image and shape space for an axial slice of a single case from the 5-fold test (Ground truth denoted as *GT*). Compare the predictions at 2*h* with the actual follow-up at 1.7*h*: With linear interpolation of the segmentations, the core area Ŝ_*c*_ remains unchanged while the huge penumbra area is faded in. Note that for a normalization of 24*h*, the fading over time would progress even slower. With the CAE and its interpolation in shape space, the shape grows non-linearly, first locally, and then quickly into the surrounding tissue-at-risk areas segmented by the U-Net in Ŝ_*cp*_.

Interpolating between the latent shape representations of core and core+penumbra estimated by the U-Net is less accurate than performing this with the latent representations of ground truth segmentations. Nevertheless, the advantages of our proposed U-Net + CAE architecture with a Dice score of 0.43 for the lesion are evident as the result is close to the ground truth interpolation (Dice 0.46). A significant improvement is found in comparison to the two baseline methods (0.36 and 0.34). It can be visually observed in Figure 7 that our linear interpolation in the shape space leads to a non-linear growth of the infarct shapes: first locally, then into the outer penumbra. Contrary to that, simply interpolating linearly on segmentation predictions leads to implausible fading of the entire tissue-at-risk infarct probabilities in the image voxel space, where the rate of progression is also strictly depending on the normalization value!

**Figure 8:**
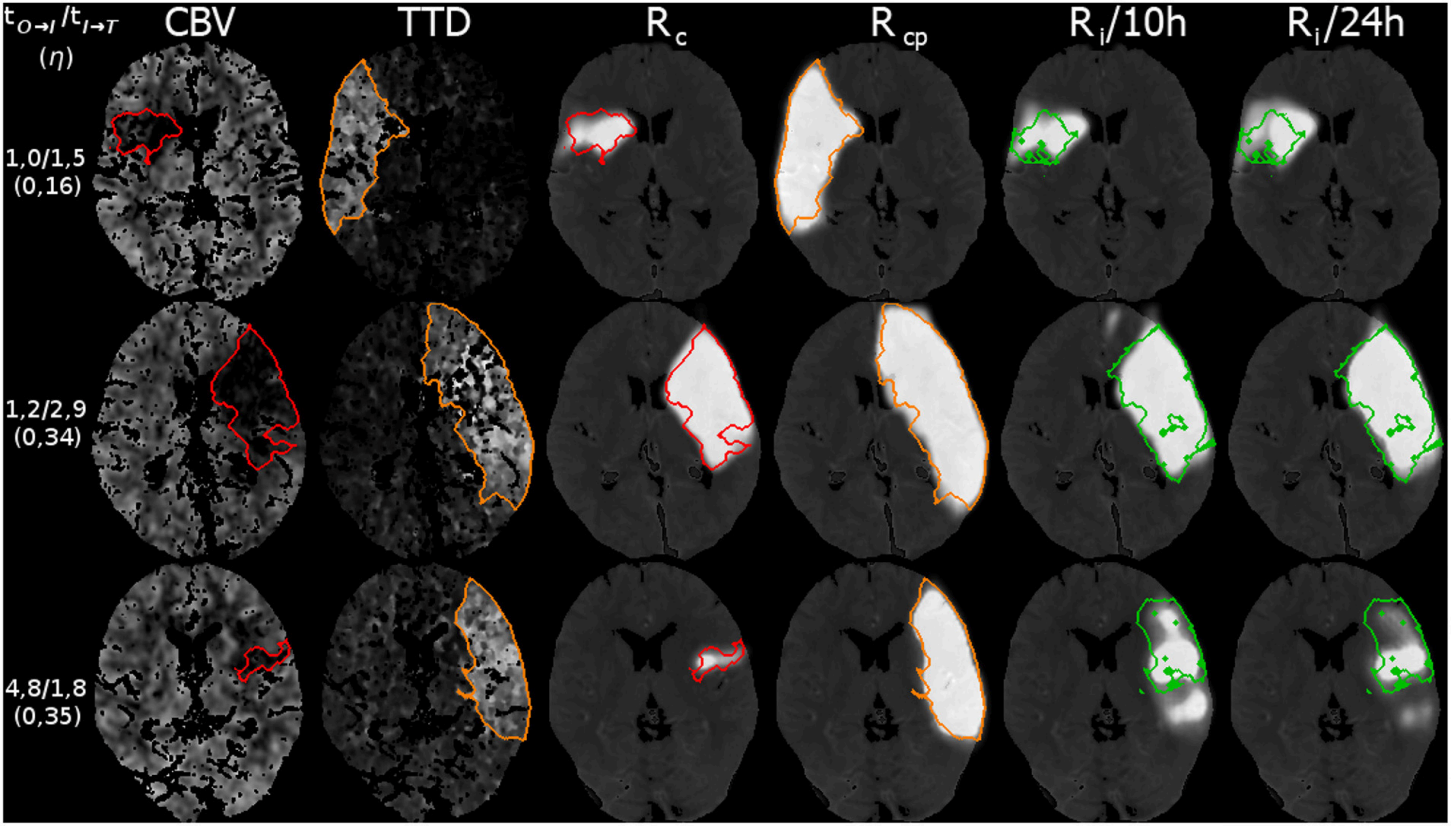
Three example cases (as in Figure 9) from the 5-fold test data with their results for the reconstruction. The top row shows a case with fast admission and treatment times, where the lesion appears about the same size as the initial core. The middle and bottom row are interpolated at η located one third on the trajectory from core to core+penumbra, while one case is an early and the other is a late admission; However, the case with bigger core progresses slowly with respect to the non-linear reconstruction compared to the smaller core case at the bottom. Reference overlays for manual segmentations *S*_*c*_ (red), *S*_*cp*_ (orange), and *S*_*l*_ (green) are shown as outlines. Note that models trained with a normalization of 10 or 24 h output similar predictions.

**Figure 9:**
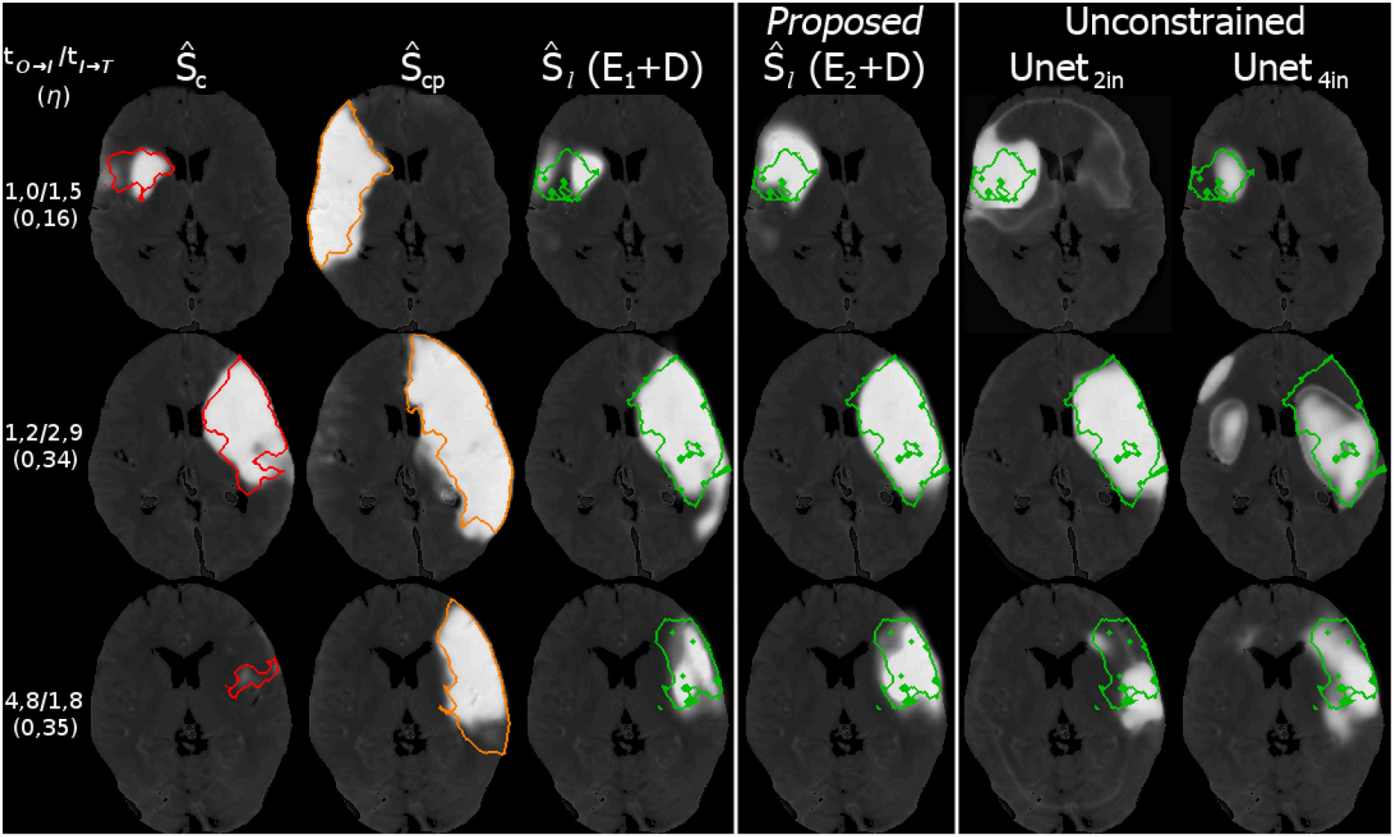
Three example cases (as in Figure 8) from the 5-fold test data with their results for the prediction. The top row shows a case with fast admission and treatment times, where the lesion appears about the same size as the initial core. The middle and bottom row are interpolated at η located one third on the trajectory from core to core+penumbra, while one case is an early and the other is a late admission; However, the case with bigger core progresses slowly with respect to the non-linear reconstruction compared to the smaller core case at the bottom. Reference overlays for manual segmentations *S*_*c*_ (red), *S*_*cp*_ (orange), and *S*_*l*_ (green) are shown as outlines. Note that prediction with unconstrained U-Net-only variants with (4in) or without (2in) clinical input channels cannot reach the prediction performance of our proposed U-Net + CAE method.

Moreover, our subdivided approach clearly reveals the sub-tasks that need to be tuned in our method for improvement of the final prediction, different to a closed unconstrained model like the single U-Net. We observed that there is less overlap for the core than for both core+penumbra. By improving the core reconstruction from shape space, the interpolation trajectory would be closer to the true lesion representation. Thus, prediction for the true time-to-treatment could benefit, for both ground truth and estimated segmentations. If furthermore the core estimate could be more accurate, the closer will the latent code of ŷ_*c*_ be located to the representation *y*_*c*_ of the ground truth core, and so will the trajectory in shape space be more close to the ideal trajectory.

Compared to the results of the ISLES stroke lesion challenges of the last 2 years on MR perfusion and diffusion data, none of the participating groups has reached a higher overlap of the predicted lesion outcome with the actual follow-up than a Dice of 0.32 (see https://www.isles-challenge.org). With respect to the similar task and comparable functional imaging modalities, the results of our method predicting the lesion outcome on core and penumbra estimates are promising.

## 6. Conclusion

In this work we have shown the feasibility of using interpolations between low-dimensional shape embeddings of core and penumbra segmentations for improving the prediction of stroke lesion tissue outcome. First, we could show that a CAE is able to model the main variances of volumetric stroke shapes resulting in good reconstructions on test data. With the latent representation at hand, one can now continuously interpolate along robust linear trajectories in the shape space to obtain non-linear shape growth from the core to the entire penumbral area. Fed with an actual time-to-treatment point, this results in a shape-constrained estimate of the expected final lesion for the given time, making it possible to compute other measures on this result, such as volume or density, to be of further assistance to the radiologist. Thus, our framework facilitates the assessment of potential infarct growth and possible salvageable tissue to support treatment decisions and prioritization.

With our current interpolation method we have an upper bound for the prediction Dice score of 0.53, which can be achieved on manual expert segmentations as end points for the linear interpolation in shape space when using a time-to-treatment oracle. This is nearly reached with our best performing fully-automatic model based on the actual time-to-treatment (Dice score 0.43). To improve the overall performance for the prediction by interpolating between the shape representations of automated core and penumbra segmentations according to time, we believe that time as a factor for the stroke growth will not always be in fixed linear relationship with the interpolation. First of all, there are other clinical variables that have an (combined) effect on the outcome, such as age or NIHSS (National Institutes of Health Stroke Scale) score, not yet considered in our method. Furthermore, differences in the growth rate even for similar early lesions could be found between patients. In future, we would like to investigate how an integrated approach can also learn non-linear growth over time to further close the gap from 0.43 to 0.53. Nevertheless, our method does not only strive for ideal overlap but rather robust growth over time in a plausible manner.

We observed that there are still cases lowering the overall prediction performance, where the follow-up lesion remains smaller than the core area. This cannot only be explained by different treatment outcomes or a decline in swelling, and requires a review with clinical experts on the dataset (perfusion parameters, manual segmentation protocol) as well as our hypothesis. For instance, if manual segmentations are not consistent throughout the dataset, rejecting data cases, which do not fit the hypothesis and thus make it difficult to train our proposed network according to our preconditions, could show substantial improvements in the results.

## Ethics statement

This study was carried out in accordance with the recommendations of the “Antrag an die Ethik-Kommsion vom 14. April 2015, Retrospektive Auswertung von CT- und MRT-Datensätzen zur Entwicklung eines Prädiktionsmodells bei Schlaganfallpatienten, Ethik-Kommission ÄK Hamburg.” with reference number AZ 15-113 (23 Apr 2015). The protocol was approved by the Ethik-Kommission ÄK Hamburg. All subjects gave written informed consent in accordance with the Declaration of Helsinki

## Data availability statement

The datasets for this manuscript are not publicly available because they were collected for institutional usage only. Requests to access the datasets should be directed to André Kemmling (andre.kemmling@uksh.de). The latest source code is available www.github.com/multimodallearning/stroke-prediction.

## Author contributions

CL conceived network architectures, interpolation and training scheme, conducted the literature review, prepared the manuscript, and partly preprocessed the dataset. MH and NB conceived the idea of auto-encoders for the core and penumbra interpolation and noted on the scheme proposed by CL. MH suggested literature for anatomical constraints in image analysis and commented on the work steadily. NB further suggested architecture variants for the interpolation problem. AK proposed the clinical task, defined the needs, and collected the dataset. LA conducted the manual segmentations and parts of the dataset preprocessing. AM gave valuable input on the perfusion segmentation via CNNs. All authors reviewed and approved the submission of this version of the manuscript.

### Conflict of interest statement

The authors declare that the research was conducted in the absence of any commercial or financial relationships that could be construed as a potential conflict of interest.
